# The nhppp package for simulating non-homogeneous Poisson point processes in R

**DOI:** 10.1371/journal.pone.0311311

**Published:** 2024-11-21

**Authors:** Thomas A. Trikalinos, Yuliia Sereda

**Affiliations:** 1 Center for Evidence Synthesis in Health, Brown University, Providence, RI, United States of America; 2 Department of Health Services, Policy & Practice, Brown University, Providence, RI, United States of America; 3 Department of Biostatistics, Brown University, Providence, RI, United States of America; Indian Statistical Institute, INDIA

## Abstract

We introduce the **nhppp** package for simulating events from one dimensional non-homogeneous Poisson point processes (NHPPPs) in R fast and with a small memory footprint. We developed it to facilitate the sampling of event times in discrete event and statistical simulations. The package’s functions are based on three algorithms that provably sample from a target NHPPP: the time-transformation of a homogeneous Poisson process (of intensity one) via the inverse of the integrated intensity function; the generation of a Poisson number of order statistics from a fixed density function; and the thinning of a majorizing NHPPP via an acceptance-rejection scheme. We present a study of numerical accuracy and time performance of the algorithms. We illustrate use with simple reproducible examples.

## 1 Introduction

It is often desirable to simulate series of events (stochastic point processes) so that the intensity of their occurrence varies over time. Examples include events such as the occurrence of death and occurrences of symptoms, infections, or tumors over a person’s lifetime. The non-homogeneous Poisson point process (NHPPP), which generalizes the simpler homogeneous-Poisson, Weibull, and Gompertz point processes, is a widely used model for such series of events. NHPPPs can model complicated event patterns given a suitable intensity function. They are, therefore, useful in statistical and mathematical model simulation.

An NHPPP has the properties that the number of events in all non-overlapping time intervals are independent random variables and that, within each time interval, the number of events is Poisson distributed. Thus an NHPPP is a memoryless point process. A large number of phenomena may reasonably conform with these properties.

NHPPPs have been used in the simulation analysis of queues in queuing theory and operations research [1, 2]; hospital operations [3]; ambulance services [4, 5]; traffic accidents [6]; product and network reliability [7]; and the modeling of cancer [8–11], heart disease [12], and dementia [13, 14], among other applications [15]. NHPPPs are used so widely in part because their assumptions are often plausible. For example, when modeling traffic accidents along a road, it may be plausible to assume that individual accidents are independent of each other, but they happen in some locations more often because the probability of an accident depends on local aspects of the road, such as turns, slopes, and propensity for slippery conditions. Similarly, when modeling the impact of screening strategies on colorectal cancer outcomes at the population level, it is probably plausible to assume that, for each person, the emergence of precancerous lesions (adenomas) over a time interval is independent of whether such lesions emerged in other non-overlapping time intervals. In these examples, the intensity of event occurrence over the carrier space (the probability of a traffic accident along a road; and the probability that an adenoma will emerge in a person’s colon at different ages) is captured by the NHPPP’s intensity function. An NHPPP can model complicated event patterns using intensity functions that vary over the carrier space (e.g., length of road, time).

The **nhppp** package in R contains functions for the simulation of NHPPPs over a one-dimensional carrier space, which we will take to represent time [16]. Table 1 summarizes the functions implemented in **nhppp** as of version 0.1.4. You can install the development version of **nhppp** with


*R> # install.packages("devtools")*
*R> devtools::install_github("bladder-ca/nhppp")*


or the release version with


*R> install.packages("nhppp")*


**Table 1:** Functions in nhppp.

Intensity function	Function name	Function simulates…
Constant	ppp_n()	exactly *n* events in [*a*, *b*)
	ppp_next_n()	the next *n* events in [*a*, ∞)
	ppp_sequential(), ppp_orderstat()	≥ 0 events in [*a*, *b*)
	ztppp()	≥ 1 events in [*a*, *b*)
Time-varying, special cases, non-vectorized	draw_sc_linear()	≥ 0 events in [*a*, *b*) from λ(*t*) = *α* + *βt*
	draw_sc_loglinear()	≥ 0 events in [*a*, *b*) from λ(*t*) = exp(*α* + *βt*)
	draw_sc_step()	≥ 0 events in [*a*, *b*) from piecewise constant λ(*t*) with uneven intervals
	draw_sc_step_regular()	≥ 0 events in [*a*, *b*) from piecewise constant λ(*t*) with regular intervals
	ztdraw_sc_linear()	≥1 events in [*a*, *b*) from λ(*t*) = *α* + *βt*
	ztdraw_sc_loglinear()	≥ 1 events in [*a*, *b*) from λ(*t*) = exp(*α* + *βt*)
Time varying, special cases, vectorized	vdraw_sc_step_regular()	≥ 0 events in [*a*, *b*) from piecewise constant λ(*t*) with regular intervals
	vztdraw_sc_step_regular()	≥ 1 events in [*a*, *b*) from piecewise constant λ(*t*) with regular intervals
Time-varying, general case, non-vectorized	draw()	(wrapper function)
	draw_cumulative_intensity_inversion()	≥ 0 events in [*a*, *b*) from Λ(*t*), Λ^−1^(*t*)
	draw_cumulative_intensity_orderstats()	≥ 0 events in [*a*, *b*) from Λ(*t*), Λ^−1^(*t*)
	draw_intensity()	≥ 0 events in [*a*, *b*) from λ(*t*), λ_*_(*t*)
	draw_intensity_step()	≥ 0 events in [*a*, *b*) from λ(*t*) and piecewise constant λ_*_(*t*)
	ztdraw_cumulative_intensity()	≥ 1 events in [*a*, *b*) from Λ(*t*), Λ^−1^(*t*)
	ztdraw_intensity()	≥ 1 events in [*a*, *b*) from λ(*t*), λ_*_(*t*)
	ztdraw_intensity_step()	≥ 1 events in [*a*, *b*) from λ(*t*) and piecewise constant λ_*_(*t*)
Time varying, general case, vectorized	vdraw()	(wrapper function)
	vdraw_intensity_step_regular()	≥ 0 events in [*a*, *b*) from λ(*t*) and piecewise constant λ_*_(*t*)
	vztdraw_intensity_step_regular()	≥ 1 events in [*a*, *b*) from λ(*t*) and piecewise constant λ_*_(*t*)
(Helper function)	get_step_majorizer()	(obtains piecewise constant λ_*_(*t*) from λ(*t*))

The table pertains to version 0.1.4 of **nhppp**. λ(*t*) is an intensity function, λ_*_(*t*) a majorizer function for λ(*t*), Λ(*t*) the integrated intensity function, and Λ^−1^(*t*) the inverse function (preimage) of Λ(*t*).

We review NHPPPs in Section 2 and algorithms for sampling from constant rate Poisson point processes in Section 3. We introduce the three sampling algorithms that are implemented in the package in Section 4. We discuss special functional forms for the intensity function (constant, piecewise constant, linear, and log-linear) in Section 5. We describe **nhppp** versus other R packages that can simulate from one dimensional NHPPPs in Section 6 and present a numerical study in Section 7. We summarize in Section 8.

## 2 The Poisson point process

### 2.1 Definition

The Poisson point process is a stochastic series of events on the real line. For some sequence of events, let *N*(*t*, *t* + Δ*t*) be the number of events in the interval (*t*, *t* + Δ*t*]. If for some positive intensity λ and, as Δ*t* → 0,

$$\begin{array}{c} \begin{array}{cc} \text{Pr}[N(t,t+\Delta t)=0] & =\;1-\lambda \Delta t+o(\Delta t),\\ \text{Pr}[N(t,t+\Delta t)=1] & =\;\lambda \Delta t+o(\Delta t),\\ \text{Pr}[N(t,t+\Delta t)>1] & =\;o(\Delta t),\;\text{and}\;\\ N(t,t+\Delta t) & ╨\;N(0,t), \end{array} \end{array}$$ (1)

then that sequence of events is a Poisson point process. In Eq (1), the third statement demands that events occur one at a time. The fourth statement implies that the process is memoryless: For any time *t*_0_, the behavior of the process is independent to what happened before that time.

### 2.2 Homogeneous Poisson point process and counting process

Assume that the next event after time *t*_0_ happens at time *t*_0_ + *X*. It follows from the above definition (see [17, par. 4.1]) that, for a constant λ, *X* is exponentially distributed

$$\begin{array}{c} X∼\text{Exponential}(\lambda ), \end{array}$$ (2)

and that the number of events is Poisson distributed over the compact interval (*a*, *b*], i.e.,

$$\begin{array}{c} N(a,b)∼\text{Poisson}(\lambda (b-a)). \end{array}$$ (3)

Eq (2) generates the homogeneous Poisson point process *Z*_1_ = *t*_0_ + *X*_1_, *Z*_2_ = *Z*_1_ + *X*_2_, …, where *Z*_*i*_ is the time of arrival of event *i* and *X*_*i*_ the inter-arrival times. We will use *Z*_(*j*)_ to denote the event in position *j* when events are ordered in increasing time. Eq (3) describes the corresponding (dual) counting process *N*_1_ = *N*(*t*_0_, *Z*_1_), *N*_2_ = *N*(*t*_0_, *Z*_2_), …, where *N*_*i*_ is the total number of events from time *t*_0_ to time *Z*_*i*_. The point process (the sequence [*Z*_*i*_] of event times) and the counting process (the sequence [*N*_*i*_] of cumulants) are two sides of the same coin.

Sampling from the constant rate point process in (2) is discussed in Section 3.

### 2.3 Non homogeneous Poisson point process and counting process

When the intensity function changes over time, the homogeneous Poisson point process generalizes to its non-stationary counterpart, an NHPPP, with intensity function λ(*t*) > 0. For details see reference [17], par 4.2]. Then, the number of events over the interval (*a*, *b*] becomes

$$\begin{array}{c} N(a,b)∼\text{Poisson}(\Lambda (a,b)), \end{array}$$ (4)

where $\Lambda \left(a,b\right)=\int_{a}^{b}\lambda \left(t\right)\;dt$ is the integrated intensity or cumulative intensity of the NHPPP. Eq (4) describes the counting process of the NHPPP, which in turn implies a stochastic point process—a distribution of events over time.

Here the simulation task is to sample event times from the point process that corresponds to intensity function λ(*t*), or equivalently, to the integrated intensity function $\Lambda \left(t\right)=\int_{0}^{t}\lambda \left(s\right)\;ds$ (Section 4). (With some abuse of notation, we define Λ(*t*) ≔ Λ(0, *t*) when *a* = 0.)

#### 2.3.1 A note on zero intensity processes

In (1), λ is strictly positive but in **nhppp** we allow it to be non-negative. If λ = 0, Pr[*N*(*t*, *t* + Δ*t*) = 0] = 1 and Pr[*N*(*t*, *t* + Δ*t*) ≥ 1] = 0. This means that no events occur and the stochastic point process in the interval (*t*, *t* + Δ*t*] is denegerate. Allowing λ(*t*) ≥ 0 has no bearing on the results of simulations. If

$$\begin{array}{c} \lambda \left(t\right)\{\begin{array}{c} >0,\;\text{for}\;t\in (a,b]\\ =0,\;\text{for}\;t\in (b,c]\\ >0,\;\text{for}\;t\in (c,d] \end{array} \end{array}$$

we can always ignore the middle interval in which no events happen.

### 2.4 Properties that are important for simulation

#### 2.4.1 Composability and decomposability of NHPPPs

The definition (1) implies that NHPPPs are composable [17, par. 4.2]: merging two NHPPPs with intensity functions λ_1_(*t*), λ_2_(*t*) yields a new NHPPP with intensity function λ(*t*) = λ_1_(*t*) + λ_2_(*t*). The reciprocal is also true: one can decompose an NHPPP with intensity function λ(*t*) into two NHPPPs, one with intensity function λ_1_(*t*) < λ(*t*) and one with intensity function λ_2_(*t*) = λ(*t*) − λ_1_(*t*). An induction argument extends the above to merging and decomposing three or more processes.

The composability and decomposability properties are important for simulation because they

- give the flexibility to simulate several parallel NHPPPs independently versus to merge them, simulate from the merged process, and then attribute the realized events to the component processes by assigning the *i*-th event to the *j*-th process with probability λ_*j*_(*Z*_*i*_)/λ(*Z*_*i*_), where λ(*t*) = ∑ λ_*j*_(*t*).
- motivate a general sampling algorithm (Algorithm 4, “thinning” [18]) that simulates a target NHPPP with intensity λ_1_(*t*) by first drawing events from an easy-to-sample NHPPP with intensity λ(*t*) > λ_1_(*t*), and then accepts sample *i* with probability λ_1_(*Z*_*i*_)/λ(*Z*_*i*_).

#### 2.4.2 Transformations of the time axis

Strictly monotonic transformations of the carrier space of an NHPPP yield an NHPPP [19]. Consider an NHPPP with intensity functions λ(*t*) and a strictly monotonic transformation of the time axis *u*: *t* ↦ *τ* that is differentiable once almost everywhere. On the transformed time axis the point process is an NHPPP with intensity function

$$\begin{array}{c} \rho \left(\tau \right)=\lambda \left(\tau \right)(\frac{du}{dt}{)}^{-1}. \end{array}$$ (5)

This property is important for simulation because

- it motivates the use of another general sampling algorithm (Algorithm 5, “time transformation” or “inversion”, [19]): A smart choice for *u* yields an easy to sample point process. The event times in the original time scale can be obtained as *Z*_*i*_ = *u*^−1^(*ζ*_*i*_), where *ζ*_*i*_ is the *i*-th event in the transformed time axis and *u*^−1^ is the inverse function of *u*.
- given that at least *i* events have realized in the time interval (*a*, *b*], it makes it possible to draw events *Z*_(*j*)_, *j* < *i* given event *Z*_(*i*)_. This is useful for simulating earlier events conditional on the occurrence of a subsequent event. Choosing *u*(*t*) ≔ *Z*_(*i*)_−*t* makes the time count backwards from *Z*_(*i*)_. In this reversed clock we draw as if in forward time exactly *i* − 1 events *ζ*_(1)_, *ζ*_(2)_, …, *ζ*_(*i*−1)_. Back transforming yields all preceding events.

Table 2 summarizes the common simulation tasks, such as simulating single events (at most one, exactly one), a series of events (possibly demanding the occurrence of at least one event), or the occurrence of a prior (event *i* − 1 given *Z*_(*i*)_). The **nhppp** package implements functions to simulate these tasks for general λ(*t*) or Λ(*t*).

**Table 2:** Common simulation needs in discrete event simulation.

#	Sampling task	Sampled times	Number of sampled events	Example
I	Any next event	{} or {*Z*_(1)_}	0 or 1	Single event that may (or may not) occur in the interval: death, progression from Stage I to Stage II cancer.
II	Exactly one next event	{*Z*_(1)_}	1	Single event which must occur in the interval: death from any cause in a lifetime-horizon simulation.
III	Any and all events	{} or {*Z*_(1)_, *Z*_(2)_, …}	≥ 0	Zero, one, or more events: emergence of one or more bladder tumors.
IV	At least one next event	{*Z*_(1)_, *Z*_(2)_, …}	≥ 1	One or more events: emergence of bladder tumors when simulating only patients with bladder tumors.
V	Event *i* − 1 given *Z*_(*i*)_	{*Z*_(*i*−1)_}	1	Find the previous event when simulating conditional on a future event: time of symptom onset given the time of symptom-driven diagnosis; onset of Stage I cancer given progression from Stage I to Stage II cancer.

All listed tasks involve sampling events over the interval (*a*, *b*] with known λ(*t*) or Λ(*t*).

## 3 Sampling the constant rate Poisson process

Sampling the constant rate Poisson process is straightforward. Algorithms 1 and 2 are two ways to sample event times in interval (*a*, *b*] with constant intensity λ. Algorithm 3 describes sampling event times conditional on observing at least *k* events within the interval of interest.

### 3.1 Sequential sampling

Algorithm 1 samples events sequentially, using the fact that the inter-event times *X*_*i*_ are exponentially distributed with mean λ^−1^ [17, par. 4.1]. It involves generation only of exponential random variates, which is cheap on modern hardware. To sample at most *k* events, change the condition for the while loop in line 3 to

$$\begin{array}{c} \text{while}\;t<b\;\&\;|Z|<k\;\text{do}. \end{array}$$

The package’s ppp_sequential() function implements constant-rate sequential sampling that returns a vector with zero or more event times in the interval [*a*, *b*). The range_t argument is a two-values vector with the bounds *a*, *b*. Setting the optional argument atmost1 to TRUE from its default value of FALSE returns the first event or an empty vector, depending on whether at least one event is drawn in the interval.

**Algorithm 1** Sequential sampling of events in interval (*a*, *b*] with constant intensity λ.

**Require**: *t* ∈ (*a*, *b*]

 1: *t* ← *a*

 2: Z←∅      ▹ Z is an ordered set

 3: **while** *t* < *b* **do**     ▹ Up to *k* earliest points: **while** t<b&|Z|<k **do**

 4:  *X* ← *X* ∼ Exponential(λ^−1^)      ▹ Mean-parameterized

 5:  *t* ← *t* + *X*

 6:  **if** *t* < *b* **then**

 7:   Z←Z∪{t}

 8:  **end if**

 9: **end while**

 10: **return** Z


*R> library("nhppp")*
*R> ppp_sequential(range_t = c(7, 10), rate = 1, atmost1 = FALSE)*
[1] 7.673885 8.650502 9.011229 9.407575


**nhppp** functions can accept a user provided random number stream object via the rng_stream option.


*R> library("rstream")*
*R> S <- new("rstream.mrg32k3a")*
*R> ppp_sequential(range_t = c(7, 10), rate = 1, rng_stream = S)*
[1] 8.793702


### 3.2 Sampling using order statistics

**Algorithm 2** Sampling events in interval (*a*, *b*] with constant intensity λ using order statistics.

**Require**: *t* ∈ (*a*, *b*]

 1: *N* ← *N* ∼ Poisson(λ(*b* − *a*))

 2: *t* ← *a*

 3: Z←∅      ▹ Z is an ordered set

 4: **if** N > 0 **then**

 5:  **for** *i* ∈ [*N*] **do**:

 6:   *U*_*i*_ ← *U*_*i*_ ∼ Uniform(0, 1)     ▹ Generate order statistics

 7:   $Z\leftarrow Z\cup \{a+\left(b-a\right)U_{i}\}$

 8:  **end for**

 9:  Z←sort(Z)

 10: **end if**

 11: **return** Z    ▹ Up to *k* earliest points: **return** $\left\{Z_{\left(i\right)}\;|\;i\leq k\;,Z_{\left(i\right)}\in Z\right\}$

Algorithm 2 first draws the number of events in (*a*, *b*] from a Poisson distribution. Conditional on the number of events, the event times *Z*_*i*_ are uniformly distributed over (*a*, *b*] [17, par. 4.1]. The algorithm returns the order statistics [*Z*_(*i*)_], obtained by sorting the event times [*Z*_*i*_] in ascending order. It is necessary to generate all event times to generate the order statistics. Thus, to sample at most *k* event times we should return the earliest *k* event times, and line 11 of the Algorithm would be changed to

$$\begin{array}{c} \text{return}\;\left\{Z_{\left(i\right)}\;|\;i\leq k,Z_{\left(i\right)}\in Z\right\}. \end{array}$$

The ppp_orderstat() function implements constant-rate sampling via the order-statistics algorithm.


*R> ppp_orderstat(range_t = c(3.14, 6.28), rate = 1/2)*
[1] 3.141663 5.700931


### 3.3 Sampling conditional on observing at least *m* events

**Algorithm 3** Sampling with constant intensity λ conditional that at least *m* events occurred in interval (*a*, *b*]. Relies on generating order statistics analogously to Algorithm 2.

**Require**: *t* ∈ (*a*, *b*]

 1: *N* ← *N* ∼ TruncatedPoisson_*N*≥*m*_(λ(*b* − *a*))      ▹ (*m* − 1)-truncated Poisson

 2: *t* ← *a*

 3: Z←∅     ▹ Z is an ordered set

 4: **if** N > 0 **then**

 5:  **for** *i* ∈ [*N*] **do**:

 6:   *U*_*i*_ ← *U*_*i*_ ∼ Uniform(0, 1)     ▹ Generate order statistics

 7:   $Z\leftarrow Z\cup \{a+\left(b-a\right)U_{i}\}$

 8:  **end for**

 9:  Z←sort(Z)

 10: **end if**

 11: **return** Z     ▹ Up to *k* earliest points: **return** $\left\{Z_{\left(i\right)}\;|\;i\leq k\;,Z_{\left(i\right)}\in Z\right\}$

Algorithm 3 is used to generate a point process conditional on observing at least *m* events. For example, if λ is the intensity of tumor generation, it can be used to simulate times of tumor emergence among patients with at least one (*m* = 1) tumor. To return the up to *k* earliest events, we modify line 11 the same way as for Algorithm 2. As an example, in a lifetime simulation we can sample the time of all-cause death by setting in Algorithm 3 *m* = 1, so that at least one event will happen in (*a*, *b*], and *k* = 1, to sample only the time of the first event *Z*_(1)_.

To sample exactly *m* events, change line 1 of Algorithm 3 to

$$\begin{array}{c} N\leftarrow m. \end{array}$$

Function ztppp() simulates times conditional on drawing at least one event—i.e., setting *m* = 1 in Algorithm 3 to sample from a zero truncated Poisson distribution in line 1.


*R> ztppp(range_t = c(0, 10), rate = 0.001, atmost1 = FALSE)*
[1] 4.411277


Function ppp_n() simulates times conditional on drawing exactly *m* events.


*R> ppp_n(size = 4, range_t = c(0, 10))*
[1] 1.762014 2.902897 6.751627 9.733794


## 4 The general sampling algorithms used in nhppp

The **nhppp** package uses three well known general sampling algorithms, namely thinning, time transformation or inversion, and order-statistics. These algorithms are efficiently combined to sample from special cases, including cases where the intensity function is a piecewise constant, linear, or log-linear function of time, as described in Section 5.2.

The thinning algorithm works with the intensity function λ(*t*), which is commonly available. The inversion and order statistics algorithms have smaller computational cost than the thinning algorithm, but work with the integrated intensity function Λ(*t*) and its inverse Λ^−1^(*z*), which may not be available. The generic function draw() is a wrapper function that dispatches to specialized functions depending on the provided arguments. It is useful for general tasks but the specialized functions are probably faster.


*R> l <- function(t) t*
R> L <- function(t) 0.5 * t^2
R> Li <- function(z) sqrt(2 * z)
*R> draw(*
*+    lambda = l, lambda_maj = l(10), range_t = c(5, 10),*
*+    atmost1 = FALSE, atleast1 = FALSE*
*+  ) |> head(n = 5)*
[1] 5.179473 5.374814 5.957391 5.992196 6.101935
*R> draw(*
*+    Lambda = L, Lambda_inv = Li, range_t = c(5, 10),*
*+    atmost1 = FALSE, atleast1 = FALSE*
*+  ) |> head(n = 5)*
[1] 5.219264 5.230747 5.369646 5.398531 5.618079


### 4.1 The thinning algorithm

The thinning algorithm relies on the decomposability of NHPPPs (Section 2.4) and is described in [18]. Let the target NHPPP have intensity function λ(*t*) and λ_*_(*t*) ≥ λ(*t*) for all *t* ∈ (*a*, *b*] be a majorizing intensity function. Think of the majorizing function as an easy-to-sample function which is the sum of the intensity of the target point process λ(*t*) and the intensity λ_*reject*_(*t*) of its complementary point-process,

$$\lambda_{*}\left(t\right)=\lambda \left(t\right)+\lambda_{reject}\left(t\right).$$

The acceptance-rejection scheme in Algorithm 4 generates proposal samples with intensity function λ_*_(*t*) and stochastically attributes them to the target process (to keep, with probability λ(*Z*)/λ_*_(*Z*)) or its complement.

**Algorithm 4** The thinning algorithm for sampling from λ(*t*).

**Require**:

  λ_*_(*t*) ≥ λ(*t*) ∀ *t* ∈ (*a*, ])    ▹ majorizing intensity function

$Z_{*}=\left\{Z_{i}^{*}\;|\;Z_{i}^{*}\;\text{are}\;\text{samples}\;\text{from}\;\lambda_{*}\left(t\right)\right\}$ ▹ $Z_{*}$ is an ordered set

 1: $N\leftarrow |Z_{*}|$

 2: Z←∅      ▹ Z is an ordered set

 3: **if** *N* > 0 **then**

 4:  **for** *i* ∈ [*N*] **do**:

 5:   *U*_*i*_ ← *U*_*i*_ ∼ Uniform(0, 1)

 6:   **if** $U_{i}<\lambda \left(Z_{\left(i\right)}^{*}\right)/\lambda_{*}\left(Z_{\left(i\right)}^{*}\right)$ **then**

 7:    $Z\leftarrow Z\cup \{Z_{\left(i\right)}^{*}\}$

 8:   **end if**

 9:  **end for**

 10: **end if**

 11: **return** Z     ▹ Up to *k* earliest points: **return** $\left\{Z_{\left(i\right)}\;|\;i\leq k\;,Z_{\left(i\right)}\in Z\right\}$

To sample the earliest *k* points, one can exit the for loop in lines 4–9 when *k* events have been sampled in line 7, or, alternatively, return the first up to *k* points in line 11.

A measure of the efficiency of Algorithm 4 is the proportion of samples that are accepted, which is

$$\begin{array}{c} \frac{\int_{a}^{b}\lambda (t)dt}{\int_{a}^{b}\lambda_{*}\left(t\right)dt} \end{array}$$ (6)

on average. Thus, the closer λ_*_(*t*) is to λ(*t*), the more efficient the algorithm.

In practice, λ_*_(*t*) can be chosen as one of the special cases in Section 5, for which we have fast sampling algorithms. For example, it can be a piecewise constant majorizer. Algorithm A in S1 Appendix can automatically generate a piecewise constant majorizer function for intensity functions that are monotonic and possibly non-continuous or Lipschitz continuous and possibly non-monotonic.

The **nhppp** package has functions that sample from time-varying intensity functions. The first function, draw_intensity(), expects a user-provided linear (λ_*_(*t*) = *α* + *βt*) or log-linear (λ_*_(*t*) = *e*^*α*+*βt*^) majorizer function.


R> lambda_fun <- function(t) exp(0.02 * t)
*R> draw_intensity(*
*+    lambda = lambda_fun, # linear majorizer*
*+    lambda_maj = c(intercept = 1.01, slope = 0.03),*
*+    exp_maj = FALSE, range_t = c(0, 10)*
*+  ) |> head (n = 5)*
[1] 1.310245 2.094217 2.908682 3.268384 8.007606
*R> draw_intensity(*
*+    lambda = lambda_fun, # log-linear majorizer*
*+    lambda_maj = c(intercept = 0.01, slope = 0.03),*
*+    exp_maj = TRUE, range_t = c(0, 10)*
*+  ) |> head (n = 5)*
[1] 0.3406743 0.6079479 0.8441584 2.6424551 3.3185387


The second function, draw_intensity_step(), expects a user-provided piecewise linear majorizer

$$\begin{array}{c} \lambda_{*}\left(t\right)=\{\begin{array}{cc} \lambda_{1} & \text{for}\;t\in \left[a_{1},b_{1}\right)=\left[a,b_{1}\right),\\ \cdots & \\ \lambda_{m} & \text{for}\;t\in \left[a_{m},b_{m}\right)\;\text{with}\;a_{m}=b_{m-1},\\ \cdots & \\ \lambda_{M} & \text{for}\;t\in \left[a_{M},b_{M}\right)=\left[a_{M},b\right), \end{array} \end{array}$$

which is specified as a vector of length *M* + 1 including the points $\left(a,{\left[b_{m}\right]}_{m=1}^{M}\right)$ and a vector of length *M* with the values ${\left[\lambda_{m}\right]}_{m=1}^{M}$ in each subinterval of (*a*, *b*]. For example, the following code splits the interval (0, 10] into *M* = 10 subintervals of length one. Because lambda_fun() is strictly increasing, its value at the upper bound of each subinterval is the supremum of the interval.


*R> draw_intensity_step(*
*+    lambda = lambda_fun,*
*+    lambda_maj_vector = lambda_fun(1:10), # 1:10 (10 intensity values)*
*+    times_vector = 0:10 # 0:10 (11 interval bounds)*
*+  ) |> head(n = 5)*
[1] 0.3825378 7.0822941 7.7839779 8.7766992 8.9554954


### 4.2 The time transformation or inversion algorithm

Algorithm 5 implements the time transformation or inversion algorithm from [19] and [17, par. 4.2]. As mentioned in Section 2.4, strictly monotonic transformations of the carrier space (here, time) of a Poisson point process yield another Poisson Point Process. In Eq (5), choosing the transformation *τ* = *u*(*t*) = Λ(*t*), so that $\frac{du(t)}{dt}=\lambda \left(t\right)$, results in *ρ*(*τ*) = 1.

This means (proof sketched in [17, par. 4.2]) that we can sample points from a Poisson point process with intensity one over the interval (*τ*_*a*_, *τ*_*b*_] = (Λ(*a*), Λ(*b*)]. Via a similar argument, we transform event times sampled on the transformed scale back to the original scale using *g*(*t*) = Λ^−1^(*τ*). The transformations *u*(⋅), *g*(⋅) are not unique—at least up to the group of affine transformations.

Function draw_cumulative_intensity_inversion() works with a cumulative intensity function Λ(*t*) and its inverse Λ^−1^(*z*), if available. If the inverse function is not available (argument Lambda_inv = NULL), the Brent bisection algorithm is used to invert Λ(*t*) numerically, at a performance cost [20].


R> Lambda_fun <- function(t) 50 * exp(0.02 * t) - 50
R> Lambda_inv_fun <- function(z) 50 * log((z + 50) / 50)
*R> draw_cumulative_intensity_inversion(*
*+    Lambda = Lambda_fun,*
*+    Lambda_inv = Lambda_inv_fun,*
*+    range_t = c(5, 10.5),*
*+    range_L = Lambda_fun(c(5, 10.5))*
*+  ) |> head(n = 5)*
[1]  6.458937  7.608496  9.060817  9.566278 10.076889


**Algorithm 5** The time transformation or inversion algorithm for sampling given Λ(*t*), Λ^−1^(*z*) [17, 19]. The notation PoissonProcess_1_ indicates sampling event times from a constant rate one Poisson point process.

**Require**: Λ(*t*), Λ^−1^(*z*), *t* ∈ (*a*, *b*]     ▹ Λ^−1^(*z*) possibly numerically

 1: *τ*_*a*_ ← Λ(*a*), *τ*_*b*_ ← Λ(*b*)

 2: $C\leftarrow C∼{\text{PoissonProcess}}_{1}\left(\tau_{a},\tau_{b}\right)$     ▹ From Algorithm 1 (or 3 for conditional sampling)

 3: $Z\leftarrow \Lambda^{-1}\left(C\right)$ ▹ Λ^−1^(⋅) as set function, meant elementwise

 4: **return** Z

### 4.3 The order statistics algorithm

The general order statistics algorithm (Algorithm 6) is a direct generalization of Algorithm 2. It first draws the number *N* of realized events. Conditional on *N*

$$\begin{array}{c} \begin{array}{cc} U_{\left(i\right)} & =\frac{\Lambda \left(Z_{\left(i\right)}\right)-\Lambda \left(a\right)}{\Lambda (b)-\Lambda (a)}∼\text{Uniform}\left(0,1\right),\\ Z_{\left(i\right)} & =\Lambda^{-1}(\Lambda \left(a\right)+U_{\left(i\right)}(\Lambda (b)-\Lambda (a))), \end{array} \end{array}$$ (7)

as discussed in [18]. Algorithm 6 makes the above explicit.

**Algorithm 6** The order statistics algorithm for sampling from an NHPPP given Λ(*t*), Λ^−1^(*z*).

**Require**: Λ(*t*), Λ^−1^(*z*), *t* ∈ (*a*, *b*]      ▹ Λ^−1^(*z*) possibly numerically

 1: *N* ← *N* ∼ Poisson(Λ(*b*) − Λ(*a*))

 2: *t* ← *a*

 3: Z←∅      ▹ Z is an ordered set

 4: **if** N > 0 **then**

 5:  **for** *i* ∈ [*N*] **do**:

 6:   *U*_*i*_ ← *U*_*i*_ ∼ Uniform(0, 1)      ▹ Generate order statistics

 7:   $Z\leftarrow Z\cup \{\Lambda^{-1}(\Lambda \left(a\right)+U_{i}(\Lambda (b)-\Lambda (a)))\}$

 8:  **end for**

 9:  Z←sort(Z)

 10: **end if**

 11: **return** Z      ▹ Up to *k* earliest points: **return** $\left\{Z_{\left(i\right)}\;|\;i\leq k\;,Z_{\left(i\right)}\in Z\right\}$

Sampling up to *k* earliest points means returning the up to *k* earliest event times. If Λ(*t*) is a positive linear function of time, λ is constant and Algorithm 6 becomes Algorithm 2.

To sample conditional on observing at least *m* events in the interval (*a*, *b*] see Algorithm B in S2 Appendix.

$$\begin{array}{c} N\leftarrow N∼{\text{TruncatedPoisson}}_{N\geq m}(\Lambda (b)-\Lambda (a)). \end{array}$$

Function draw_cumulative_intensity_orderstats() works with a cumulative intensity function Λ(*t*) and its inverse Λ^−1^(*z*), if available. Function ztdraw_cumulative_intensity() conditions that at least one event is sampled in the interval. As above, if the inverse function is not available (argument Lambda_inv = NULL), the Brent bisection algorithm is used to invert Λ(*t*) numerically, at a performance cost.


*R> draw_cumulative_intensity_orderstats(*
*+    Lambda = Lambda_fun,*
*+    Lambda_inv = Lambda_inv_fun,*
*+    range_t = c(4.1, 7.6)*
*+  )*
[1] 5.091581 5.526070 5.601576 5.762498 6.495684
*R> ztdraw_cumulative_intensity(*
*+    Lambda = Lambda_fun,*
*+    Lambda_inv = Lambda_inv_fun,*
*+    range_t = c(4.1, 7.6)*
*+  )*
[1] 5.063676 6.682454 6.749162 6.926164 7.298342


## 5 Special cases

The **nhppp** package implements several special cases where the intensity function λ(⋅), the integrated intensity function Λ(⋅), and its inverse Λ^−1^(⋅) have straightforward analytical expressions.

### 5.1 Sampling a piecewise constant NHPPP

Functions draw_sc_step() and draw_sc_step_regular() sample piecewise constant intensity functions based on Algorithm 5. The first can work with unequal-length subintervals (*a*_*m*_, *b*_*m*_]. The second results in a small computational time improvement when all subintervals are of equal length.


*R> draw_sc_step(*
*+    lambda_vector = 1:5, times_vector = c(0.5, 1, 2.4, 3.1, 4.9, 5.9),*
*+    atmost1 = FALSE, atleast1 = FALSE*
*+  ) |> head(n = 5)*
[1] 0.8425117 1.3281115 2.3309443 2.6794560 2.7939130
*R> draw_sc_step_regular(*
*+    lambda_vector = 1:5, range_t = c(0.5, 5.9), atmost1 = FALSE,*
*+    atleast1 = FALSE*
*+  ) |> head(n = 5)*
[1] 2.058468 2.100620 2.508954 3.125179 3.604882


Function vdraw_sc_step_regular() is a vectorized version of draw_sc_step_regular(). It returns a matrix with one event series per row, and as many columns as the maximum number of events across all draws.


*R> vdraw_sc_step_regular(*
*+    lambda_matrix = matrix(runif(20), ncol = 5), range_t = c(1, 4),*
*+    atmost1 = FALSE*
*+  )*
         [,1]     [,2]     [,3]     [,4]
[1,] 2.304123 2.802767       NA       NA
[2,] 2.990953       NA       NA       NA
[3,] 1.840374 2.134357 3.784424 3.816034
[4,] 2.136138 2.703826 3.269631       NA


The corresponding functions that return at least one event in the interval are ztdraw_sc_step(), ztdraw_sc_step_regular(), and vztdraw_sc_step_regular().

### 5.2 Sampling NHPPPs with linear and log-linear intensities

Functions draw_sc_linear() and ztdraw_sc_linear() sample zero or more and at least one event, respectively, from NHPPPs with linear intensity functions. An optional argument (atmost1) returns the first event only.

$$\begin{array}{c} \lambda \left(t\right)=\{\begin{array}{cc} \alpha +\beta t & \;\text{for}\;t\in \left[a,b\right),t>-\frac{\alpha }{\beta }\\ 0 & \text{otherwise} \end{array}. \end{array}$$

*R> draw_sc_linear(alpha = 3, beta = -0.5, range_t = c(0, 10)) |> head(n = 5)*
[1] 0.3327657 0.4270154 0.5804320 0.6935027 0.9832093
*R> ztdraw_sc_linear(alpha = 0.5, beta = 0.2, range_t = c(9.999, 10))*
[1] 9.999757


An analogous set of functions ([nhppp|ztnhppp]_sc_loglinear()) samples from log-linear intensity functions

$$\begin{array}{c} \lambda \left(t\right)=\{\begin{array}{cc} e^{\alpha +\beta t} & \;\text{for}\;t\in [a,b)\\ 0 & \text{otherwise} \end{array}. \end{array}$$

The sampling algorithm is a variation of Algorithm 5, as described in [21]. Example usage follows.


*R> draw_sc_loglinear(alpha = 1, beta = -0.02, range_t = c(8, 10))*
 [1] 8.028806 8.128887 8.457669 8.483558 8.498647 8.503109 8.522725
 [8] 8.665979 8.671737 8.978065 8.981105 9.493691 9.815000 9.909167
*R> ztdraw_sc_loglinear(alpha = 1, beta = -0.02, range_t = c(9, 10))*
[1] 9.038160 9.075722 9.238302


## 6 Comparisons with other R packages

Table 3 lists five R packages that simulate from NHPPPs, including **nhppp**. We did not consider research code that is not an R package in the Comprehensive R Archive Network or is developed in other languages. For example, we do not run comparisons with the R and Python code for sampling from piecewise constant NHPPPs with regular time intervals in Garibay *et al* [22]. (Their code corresponds to the vdraw_sc_step_regular() function in **nhppp**.)

**Table 3:** NHPPP generation in R packages.

R package	Function	Algorithms (inputs)			Sample only earliest event	Custom RNG	Simulate given *N* > 0	Vectorized functions
		Thinning	Inversion	statistics				
**nhppp**	[see text]	λ(*t*), λ_*_(*t*)	Λ(*t*), Λ^−1^(*z*)	Λ(*t*), Λ^−1^(*z*)	Yes	**rstream** objects	Yes	For piecewise constant intensity
**reda**	simEvent()	λ(*t*), λ_*_ constant	λ(*t*) (no Λ(*t*), Λ^−1^(*z*))	No	Yes	No	No	No
**simEd**	thinning()	λ(*t*), ${\left[\lambda_{*m}\right]}_{m=1}^{M}$	No	No	No	No	No	No
**IndTestPP**	simNHPc()	${\left[\lambda_{m}\right]}_{m=1}^{M}$ , λ_*_ constant	${\left[\lambda_{m}\right]}_{m=1}^{M}$ (no Λ(*t*), Λ^−1^(*z*))	No	No	No	No	No
**NHPoisson**	simNHP.fun()	No	λ(*t*), (no Λ(*t*), Λ^−1^(*z*))	No	No	No	No	No

RNG: random number generator object.

Package **reda** [23] focuses on recurrent event data analysis and can simulate NHPPPs with the inversion and thinning algorithms using the simEvent() function. It can take function object arguments for λ(*t*). When using the thinning algorithm, it takes a constant majorizer. For the inversion algorithm, it approximates Λ(*t*) and its inverse numerically, at a computational cost.

Package **simEd** [24] includes various functions for simulation education. Function thinning() implements the homonymous algorithm for drawing points from an NHPPP. Users can specify the intensity function and a piecewise constant or linear majorizer function.

Package **IndTestPP** [25] provides a framework for exploring the dependence between two or more realizations of point processes. It includes the ancillary function simNHPc() for simulating NHPPPs with the inversion or thinning algorithms. The function’s argument is a piecewise constant approximation of the intensity function via a vector of evaluations, each corresponding to unit length subintervals. This resolution may not be adequate to simulate processes that change fast over a unit time interval.

Package **NHPoisson** [26, 27] fits NHPPP models to data and is not really geared towards mathematical simulation. Its simNHP.fun() function provides the ability for simulation-based inference via an implementation of the inversion algorithm. This function is designed to work with the package’s inference machinery and is not practical to use for simulation, because the user has no direct control over the function’s rescaling of the time axis.

The claimed advantage of **nhppp** over the existing packages is that

- it samples from the target NHPPP and not from a numerical approximation thereof, e.g., as **IndTestPP** does.
- It can sample conditional on observing at least one event in the interval, which no other package implement.
- It accepts user-provided random number stream objects, which is useful for implementing simulation variance reduction techniques such as common random numbers [28] and antithetic variates [29].
- It is fast and memory efficient, both for the non-vectorized functions that are implemented in native R and for the vectorized functions that use C++ plugins via the **Rcpp** package [30]. **nhppp** has specialized functions to leverage additional information about the point process, such as Λ(*t*), Λ^−1^(*z*), when available, which can result in faster simulation use the cumulative intensity function and its inverse, often at a computational speed advantage.

## 7 Illustrations

Depending on the application, we may have access to the intensity function or the integrated intensity function. We compared the R packages in Table 3 for sampling from a non-monotonic and highly non-linear intensity function for which the integrated intensity function can be derived analytically.

### 7.1 The target NHPPP to be simulated

Consider the example

$$\begin{array}{c} \begin{array}{cc} \lambda (t) & =e^{rt}\left(1+\text{sin}\;wt\right),\\ \Lambda (t) & =\frac{e^{rt}\left(r\;\text{sin}\;wt-w\;\text{cos}\;wt\right)+w}{r^{2}+w^{2}}+\frac{e^{rt}-1}{r} \end{array} \end{array}$$ (8)

of a sinusoidal intensity function λ(*t*) scaled to have an exponential amplitude and one of its antiderivatives Λ(*t*), with such a constant term that Λ(0) = 0. For the numerical study we set *r* = 0.2, *w* = 1, and *t* ∈ (0, 6*π*]. There is no analytic inverse function for this example. However, we can precompute Li(), a good numerical approximation to Λ^−1^(*z*). We will use it in Section 7.5 to compare the time performance of functions that use the inversion and order statistics algorithms when Λ^−1^ is available versus not.


R> l <- function(t) (1 + sin(t)) * exp(0.2 * t)
*R> L <- function(t) {*
+    exp(0.2 * t) * (0.2 * sin(t) - cos(t)) / 1.04 +
+      exp(0.2 * t) / 0.2 - 4.038462
*+  }*
*R> Li <- approxfun(*
+    x = L(seq(0, 6 * pi, 10^-3)),
+    y = seq(0, 6 * pi, 10^-3), rule = 2
*+  )*


**Fig 1:**
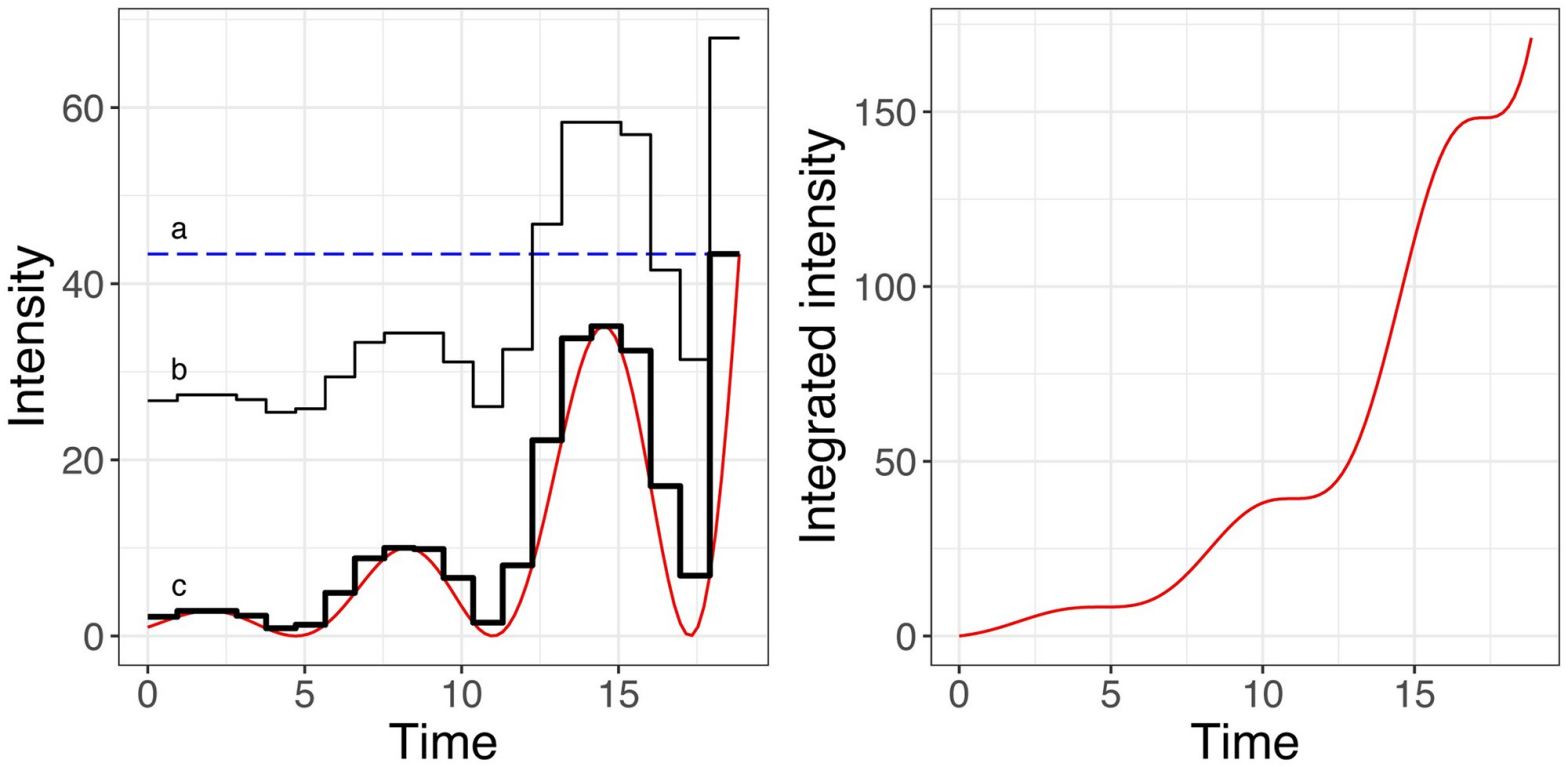
The λ(*t*) (left) and Λ(*t*) used in the illustration. Also shown three majorizing functions (left panel, marked a, b, c) that are used with the thinning algorithm in the analyses.

Fig 1 graphs the intensity function and three majorizing functions over the interval of interest, which will be needed for the thinning algorithm.

The first, λ_*a_(*t*) = 43.38, shown as a dashed blue line, is is a constant majorizer equal to the maximum of the intensity function. A constant majorizer may be a practical choice when only an upper bound is known for λ(*t*). From (6), the analytic efficiency of the thinning algorithm using this majorizer is 0.209.

The second, λ_*b_(*t*), shown as a thin black line, is a piecewise constant envelope generated automatically from Algorithm A in S1 Appendix with 20 equal-length subintervals and Lipschitz cone coefficient *K* = 52.05. We set *K* equal to the maximum value of $|\frac{d\lambda (t)}{dt}|$ in the interval, attained at 6*π*. The analytic efficiency of the thinning algorithm using this majorizer is 0.245.

The third, λ_*c_(*t*), shown as a thicker black line, is a tighter piecewise constant majorizer with the same 20 equal-length subintervals that is constructed by finding a least upper bound in each subinterval. The analytic efficiency of the thinning algorithm with the third majorizer is 0.718.

### 7.2 Simulation functions and algorithms

We sampled series of events from the target NHPPP using the packages and functions listed in Table 3. We repeated the sampling 10^4^ times, recording all simulated points (event times). We also recorded the median computation time for drawing one series of events with single-threaded computation on modern hardware.

From the **nhppp** package we use

1. two functions that take as argument the intensity function and are based on Algorithm 4 (thinning): draw_intensity(), which uses linear majorizers such as λ_*a_, and draw_intensity_step(), which uses piecewise constant majorizers such as λ_*b_ and λ_*c_ in the example.
2. Function draw_cumulative_intensity_inversion(), which takes as argument the cumulative intensity function Λ(*t*) and is based on Algorithm 5 (time transformation/inversion), and
3. function draw_cumulative_intensity_orderstats(), which also uses Λ(*t*) and is based on Algorithm 6 (order statistics).

Regarding the other R packages in Table 3, we used all except for **NHPoisson**, whose simulation function is tailored to supporting simulation based inference for data analysis and is not practical to use as a standalone function. (Its implementation does not allow the user to control the scaling of the time axis in a practical way.) However, its source code/algorithm is very similar to that of the **IndTestPP** simulation function, which is developed by the same authors.

We used the metrics in Table 4 to assess simulation performance with each function. We compared the empirical versus the simulated distributions of number of events and event times over *J* = 100 simulation runs.

**Table 4:** Simulation metrics for the number of counts.

Metric	Definition	Description
Bias in mean	$B_{\mu }=\frac{1}{J}\sum_{j}n_{j}-N$	Mean difference from target in the number of counts.
Relative bias in mean	$B_{\mu ,rel}=\frac{B_{\mu }}{N}$	Mean proportional difference from target in the number of counts.
Bias in variance	$B_{V}=\frac{1}{J}\sum_{j}(n_{j}-\frac{1}{J}\sum_{j}n_{j}{)}^{2}-V$	Mean difference from target in variance of counts.
Relative bias in variance	$B_{V,rel}=\frac{B_{V}}{V}$	Mean proportional difference from target in variance of counts.
Equal-tailed *p*% confidence interval bounds	*n*_[*p*/2]_, *n*_[1−*p*/2]_	Quantiles of the empirical distribution of counts.
Goodness of fit *p* value	Statistic $\sum_{x}\frac{{\left(O_{x}-E_{x}\right)}^{2}}{E_{x}}∼\chi_{U-L+1}^{2}$	Left-tail *p* value. *p* values near 1 imply good fit.
Wasserstein-1 distance	*W*_1_, the smallest rearrangement of probability mass so that one distribution matches the other.	*W*_1_ = 0 implies good fit
*p* value for *W*_1_ ≠ 0	Asymptotic theory *p* value	Two-sided *p* value. *p* values near 1 imply good fit.

In the Table, *j* ∈ [*J*] indexes simulations, *n*_*j*_ is the number of counts in simulation *j*, *N* = Λ(6*π*) − Λ(0) is the theoretical mean number of counts, and *V* = Λ(6*π*) − Λ(0) = *N* the theoretical variance. The lower and upper bounds of an equal-tailed *p*% confidence interval, *p* ∈ {95, 90, 75, 50}, are denoted with *n*_[*p*/2]_, *n*_[1−*p*/2]_, respectively. For the goodness of fit, we created bins [0, *L*), [*L*, *L* + 1), …, [*U*, ∞), where *L*, *U* are the 0.001 and 0.999 percentiles of the Poisson distribution with parameter Λ(6*π*) − Λ(0). We indexed bins with *x* ∈ {1, …, *U* − *L* + 2}. The goodness of fit statistic contrasts the observed (*O*_*x*_) versus expected (*E*_*x*_) numbers of events over the bins and it is compared with a $\chi_{U-L+1}^{2}$ distribution to obtain a *p* value.

### 7.3 Simulation performance with respect to number of events

We calculated the absolute and relative bias in the first two moments of the empirical distribution in the counts of events, the bounds of equal-tailed confidence intervals at the 95, 90, 75, and 50 percent levels, a *χ*^2^-distributed goodness of fit statistic and its *p*-value, and the Wasserstein-1 distance *W*_1_ between the empirical and the theoretical count distributions and the asymptotic one sided *p* value to reject whether *W*_1_ = 0 according to [31]. *W*_1_ is the smallest mass that has to be redistributed so that one distribution matches the other. *W*_1_ is equal to the unsigned area between the cumulative distribution functions of the compared distributions. For example, *W*_1_ = 5.25 means that the mass that must be moved to transform one density to the other is no less than 5.25 counts and a *W*_1_ = 0 implies perfect fit.

**Fig 2:**
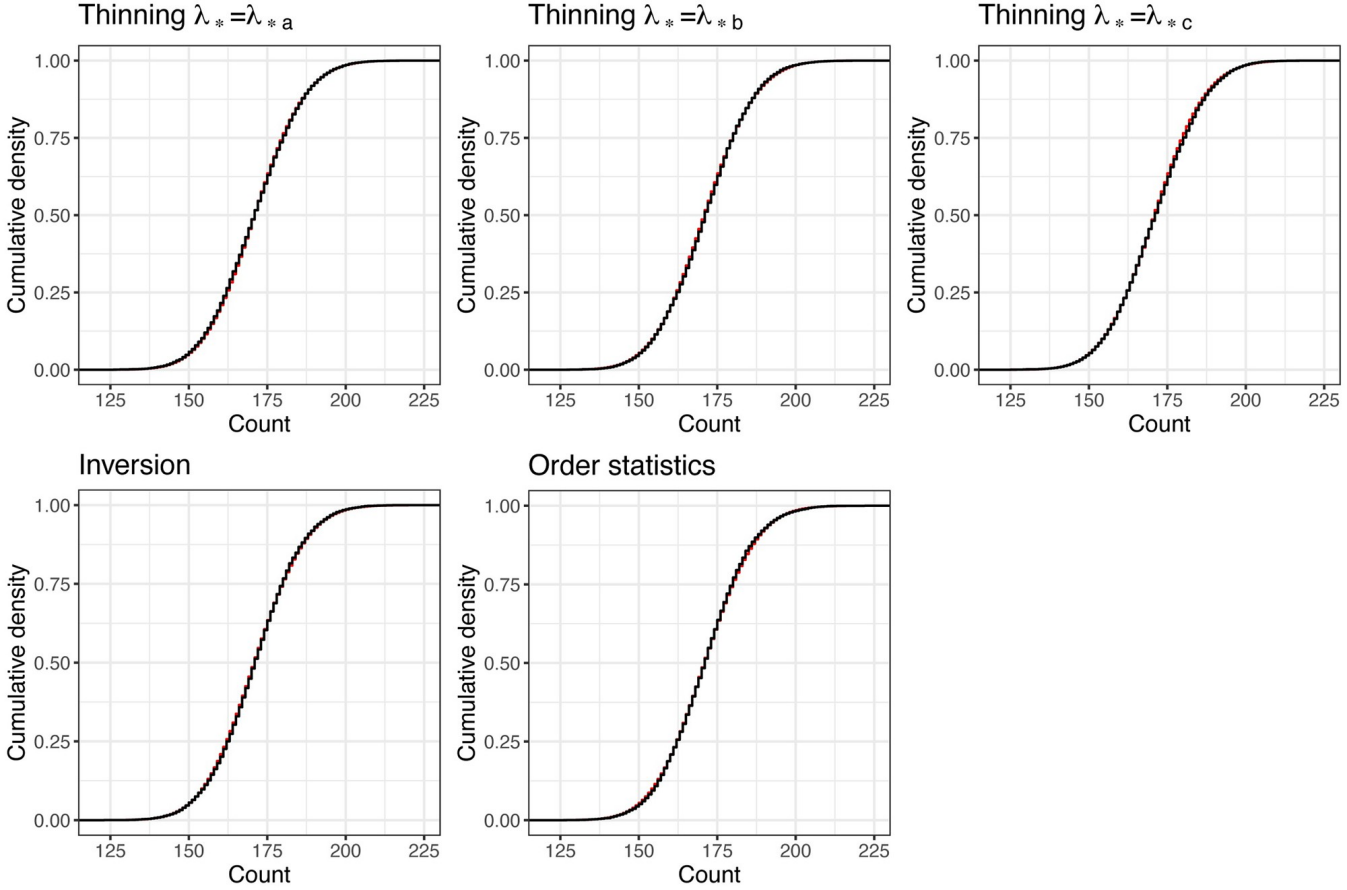
Theoretical (red) and empirical (black) cumulative distribution functions for event counts in the illustration example with nhppp functions. The unsigned area between the theoretical and empirical curves equals the Wasserstein-1 distance in Table 5.

The results for the **nhppp** functions in Fig 2 and Table 5 suggest excellent simulation performance.

**Table 5:** Simulated total number of events with nhppp functions for the illustration example.

	Thinning λ_*_=λ_*a_	Thinning λ_*_=λ_*b_	Thinning λ_*_=λ_*c_	Inversion	Order statistics
Sample mean	171.057	171.257	171.322	171.193	171.131
*B*_*μ*_	-0.078	0.122	0.187	0.058	-0.004
*B*_*μ*,*rel*_	-0.045	0.071	0.109	0.034	-0.002
Sample variance	175.015	168.218	173.918	166.950	166.933
*B*_*V*_	3.880	-2.917	2.783	-4.185	-4.201
*B*_*V*,*rel*_	2.267	-1.704	1.626	-2.445	-2.455
Goodness of fit, *χ*^2^ [*p* value]	0.145 [1.000]	0.160 [1.000]	0.117 [1.000]	0.384 [1.000]	0.229 [1.000]
*W*_1_ [*p* value]	0.194 [1.000]	0.189 [1.000]	0.231 [0.997]	0.195 [1.000]	0.187 [1.000]
Equal tail 95% CI = [146, 197]	[146, 197]	[146, 197]	[146, 197]	[146, 197]	[146, 197]
Equal tail 90% CI = [150, 193]	[150, 193]	[150, 193]	[150, 193]	[150, 193]	[150, 193]
Equal tail 75% CI = [156, 186]	[156, 186]	[156, 186]	[156, 187]	[156, 186]	[156, 186]
Equal tail 50% CI = [162, 180]	[162, 180]	[162, 180]	[162, 180]	[162, 180]	[162, 180]

Equal tail *p*% CI: a confidence interval whose bounds are the *p*/2 and (1 − *p*/2) count percentiles of the respective cumulative distribution function.

**Fig 3:**
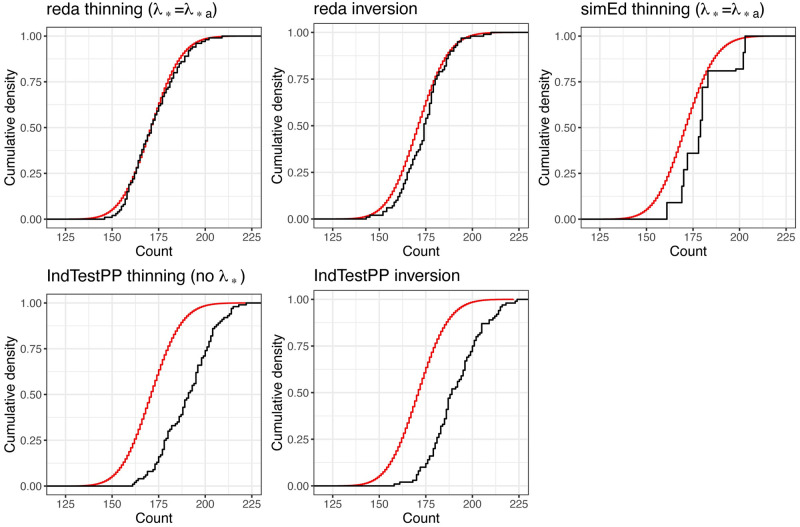
Theoretical (red) and empirical (black) cumulative distribution functions for event counts in the illustration example with the R packages in Table 3. The unsigned area between the theoretical and empirical curves equals the Wasserstein-1 distance in Table 5.

The respective results for the R packages are in Fig 3 and Table 6. The simulation performance with the **reda** functions is excellent. Performance with **simEd** and **IndTestPP** functions depends on the adequacy with which they approximate the target density. In this example, the approximation accuracy is not ideal for either package, but is somewhat worse for **IndTestPP**.

**Table 6:** Simulated total number of events with the R packages of Table 3 for the illustration example.

	reda thinning, λ_*_=λ_*a_	reda inversion	simEd thinning, λ_*_=λ_*a_	IndTestPP thinning, no λ_*_	IndTestPP inversion
Sample mean	172.430	174.170	179.910	190.490	191.030
*B*_*μ*_	1.295	3.035	8.775	19.355	19.895
*B*_*μ*,*rel*_	0.757	1.774	5.128	11.310	11.626
Sample variance	168.429	145.193	155.355	194.838	191.484
*B*_*V*_	-2.705	-25.942	-15.779	23.704	20.349
*B*_*V*,*rel*_	-1.581	-15.159	-9.220	13.851	11.891
Goodness of fit, *χ*^2^ [*p* value]	6.830 [1.000]	10.720 [1.000]	67.482 [0.994]	226.107 [<0.001]	237.199 [<0.001]
*W*_1_ [*p* value]	1.453 [0.256]	3.083 [0.112]	8.856 [<0.001]	19.356 [0.086]	19.896 [0.170]
Equal tail 95% CI = [146, 197]	[152, 199]	[152, 196]	[161, 203]	[163, 214]	[168, 217]
Equal tail 90% CI = [150, 193]	[154, 195]	[154, 192]	[161, 203]	[167, 214]	[170, 215]
Equal tail 75% CI = [156, 186]	[158, 189]	[161, 187]	[169, 202]	[174, 205]	[176, 207]
Equal tail 50% CI = [162, 180]	[162, 181]	[165, 180]	[170, 183]	[178, 201]	[181, 200]

Equal tail *p*% CI: a confidence interval whose bounds are the *p*/2 and (1 − *p*/2) count percentiles of the respective cumulative distribution function.

### 7.4 Event times

We compared the theoretical and empirical distribution of event times for all *J* = 10^4^ event time draws. We calculated a goodness of fit statistic by binning realized times in 70 bins and its *p* value, by comparing the statistic against the $\chi_{69}^{2}$ distribution. We also calculated the *W*_1_ distance between these distributions and its associated *p* value.

**Fig 4:**
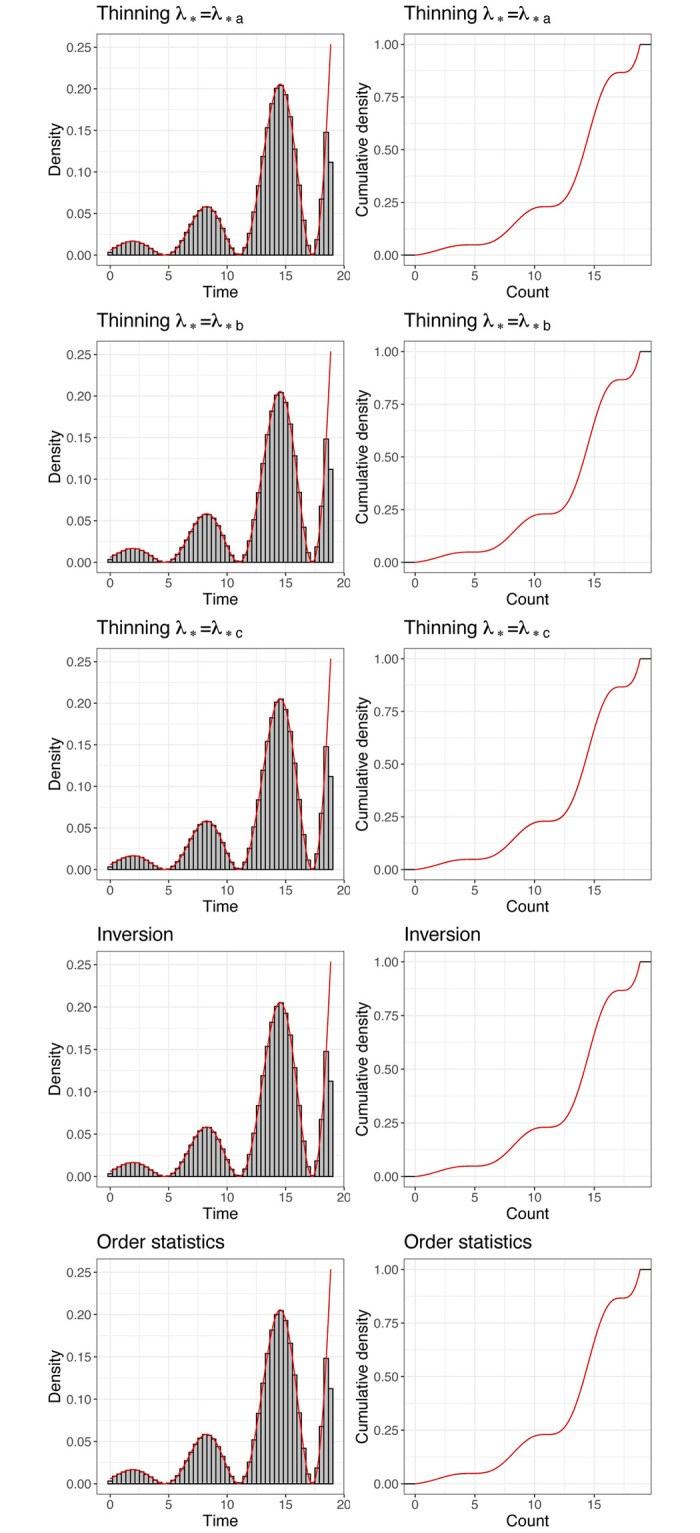
Simulated event times with nhppp. Left column: histogram (gray) and theoretical distribution (red) of event times; right column: empirical (black) and theoretical (red) cumulative distribution function. The unsigned area between the empirical and cumulative distribution functions is the *W*_1_ distance in Table 7.

Fig 4 and Table 7 indicate excellent simulation performance with the **nhppp** functions.

**Table 7:** Goodness of fit of simulated event times with nhppp functions for the example.

	Goodness of fit, *χ*^2^ [*p* value]	*W*_1_ [*p* value]
Thinning λ_*_=λ_*a_	0.004 [1.000]	0.396 [1.000]
Thinning λ_*_=λ_*b_	0.004 [1.000]	0.361 [1.000]
Thinning λ_*_=λ_*c_	0.004 [1.000]	0.338 [1.000]
Inversion	0.004 [1.000]	0.347 [1.000]
Order statistics	0.004 [1.000]	0.350 [1.000]

**Fig 5:**
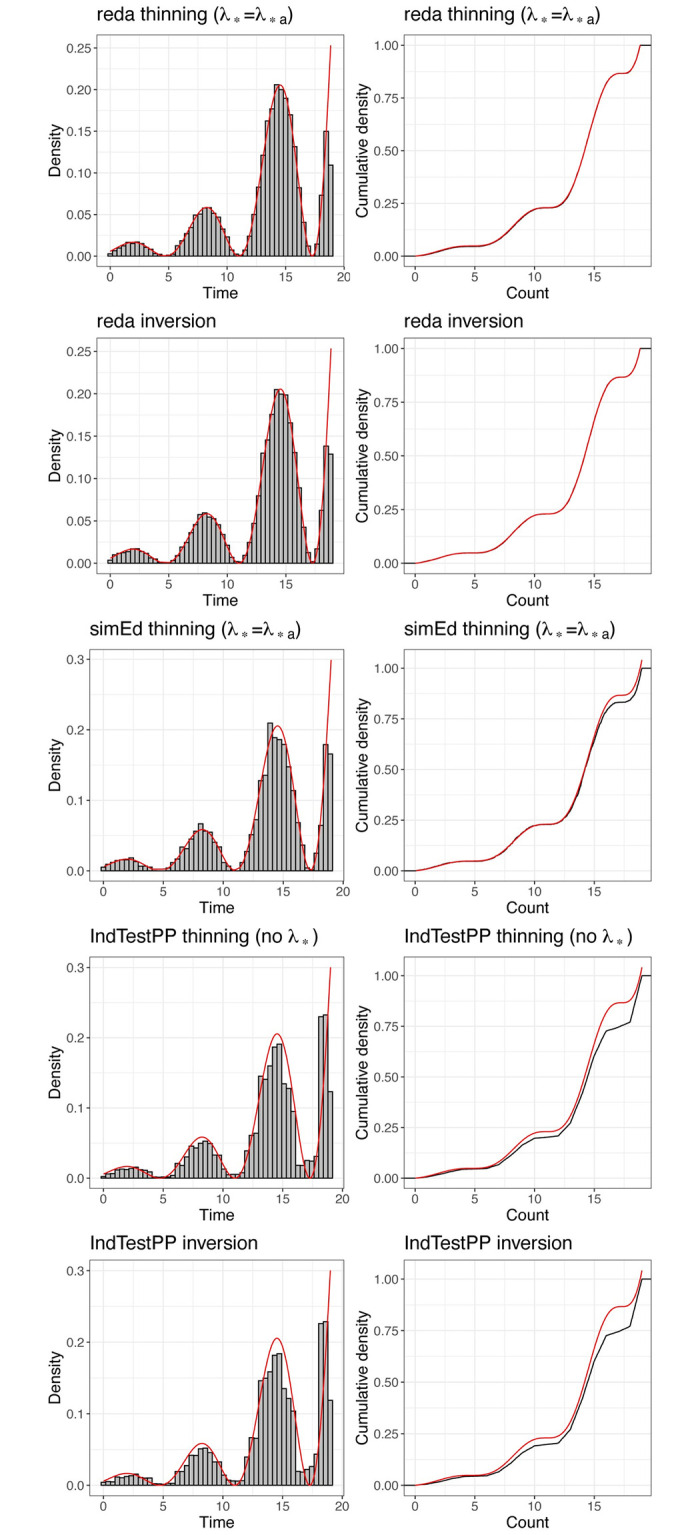
Simulated event times with the R packages in Table 3. Left column: histogram (gray) and theoretical distribution (red) of event times; right column: empirical (black) and theoretical (red) cumulative distribution function. The unsigned area between the empirical and cumulative distribution functions is the *W*_1_ distance in Table 8.

Fig 5 and Table 8 indicate excellent simulation performance with the **reda** functions. The simulation performance with the **simEd** and **IndTestPP** functions, which rely on approximations, is not as good.

**Table 8:** Goodness of fit of simulated event times with R functions in Table 3.

	Goodness of fit, *χ*^2^ [*p* value]	*W*_1_ [*p* value]
**reda** thinning (λ_*_=λ_*a_)	0.012 [1.000]	0.356 [1.000]
**reda** inversion	0.010 [1.000]	0.354 [1.000]
**simEd** thinning (λ_*_=λ_*a_)	0.028 [1.000]	0.338 [0.990]
**IndTestPP** thinning (no λ_*_)	0.460 [1.000]	2.152 [0.930]
**IndTestPP** inversion	0.490 [1.000]	2.372 [0.927]

### 7.5 Time performance

#### 7.5.1 Time performance of non-vectorized functions

**Fig 6:**
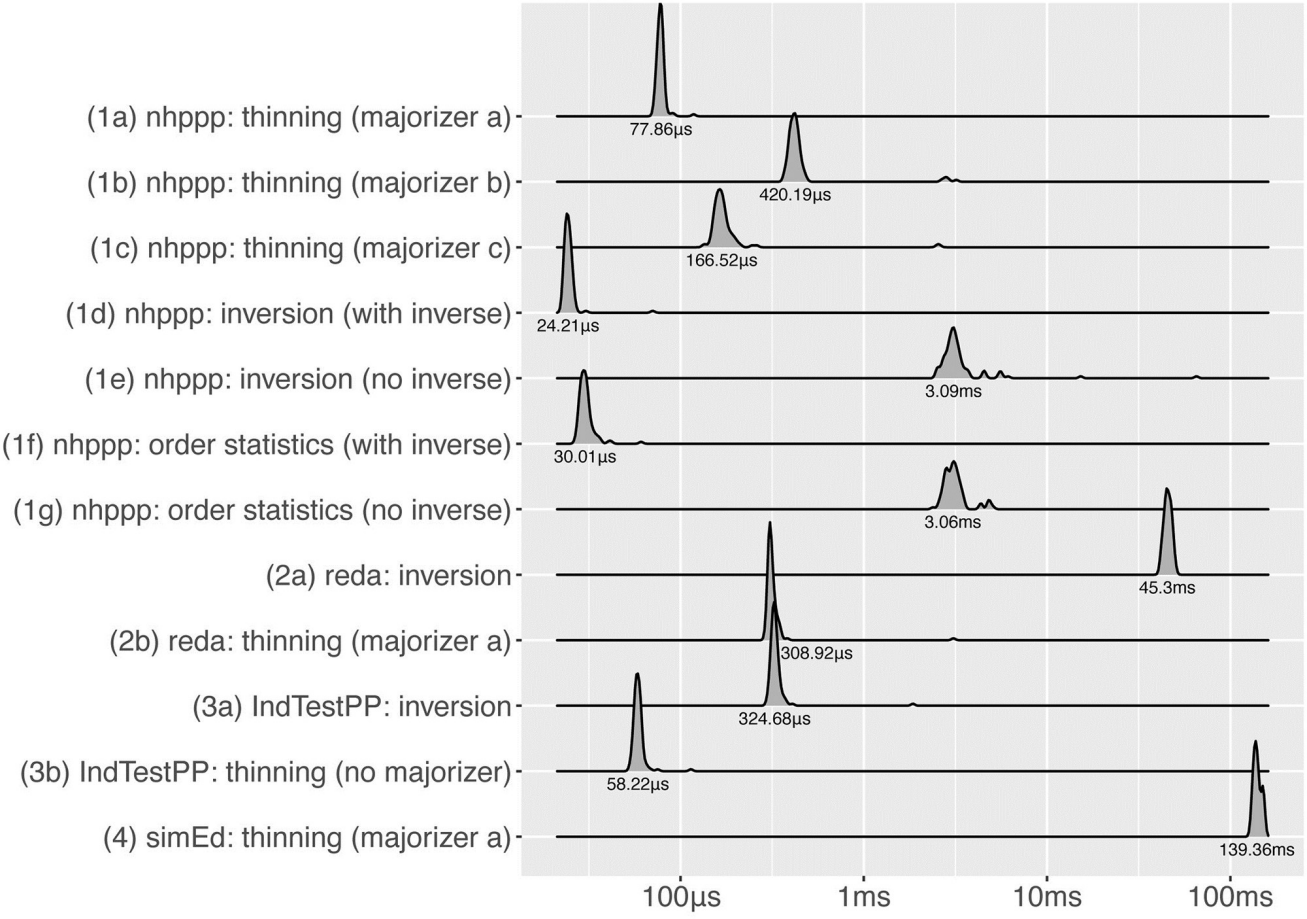
Computation times when drawing all events in interval.

**Fig 7:**
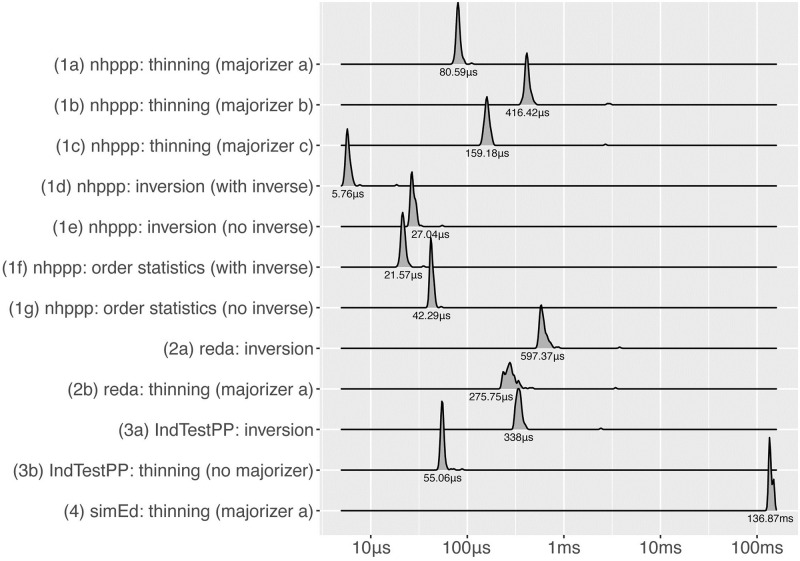
Computation times when drawing the first event in interval.

To indicate time performance, we benchmarked functions by recording execution times when drawing a series of points (Fig 6). We also benchmarked functions for drawing the first-occurring event, because **nhppp** functions can sample the first time more efficiently when the inversion algorithm is used (Fig 7).

We provided functions with the arguments they need to run fastest. For example, functions that use the inversion or order statistics algorithm execute faster when the inverse function Λ^−1^(*z*) is provided, rather than numerically calculated, as shown in both Figures for the **nhppp** package. (Functions in other packages do not take Λ(*t*) and Λ^−1^(*z*) arguments.) The fastest functions are **nhppp** functions that rely on the inversion or order statistics algorithms given Λ^−1^(*z*).

According to (6), the thinning algorithm has higher efficiency, and is expected to execute faster, for majorizer functions that envelop the intensity function more closely. Observe that λ_*a_ ≻ λ_*c_ and λ_*b_ ≻ λ_*c_ in Fig 1. As expected, the execution times are indeed shorter for majorizer ‘c’ compared to ‘b’ in Figs 6 and 7. However, the execution times are longer with majorizer ‘c’ compared to ‘a’ because draw_intensity(), the function that uses constant majorizers, and draw_intensity_step(), the function that use piecewise constant majorizers, are implemented differently. draw_intensity() happens to be faster in this example, but this is not always true.

In **nhppp**, functions that use the inversion or order statistics algorithms can exit earlier when only the first event is requested. This is not possible, however, for the thinning algorithm. This efficiency does not appear to be implemented in the other packages.

#### 7.5.2 Time performance of vectorized functions

In R, ‘vectorized’ computation, where operations are done in columns, is faster than using for loops or apply() functions. As shown in Table 1, **nhppp** includes vectorized functions for sampling from (i) piecewise constant intensity functions, using [vdraw|vztdraw]_sc_step_regular(); and (ii) general intensity functions, using [vdraw|vztdraw]_intensity_step_regular().

We compared the execution speed of non-vectorized and vectorized functions for sampling 10^5^ times from the piecewise constant ‘b’ majorizer (λ_*b_) in Fig 1. The expected number of events with λ_*b_ in (0, 6*π*] is 741.97. When drawing only the earliest event, the vectorized function is approximately 113 times faster than the non-vectorized function (median 59*ms* versus 6717*ms* over 10^5^ simulations). When drawing all events, the vectorized function is approximately 1.4 times faster than the non-vectorized function (median 36.55*s* versus 50.97*s* over 10^5^ simulations). The reason that the difference in speed attenuates is that the current implementation of the vectorized functions does not use sparse matrices to store samples, which introduces inefficiencies the expected number of samples becomes larger.

## 8 Summary and next developments

The **nhppp** facilitates the simulation of NHPPPs from time-varying intensity or cumulative intensity functions. Its claim is that it (i) simulates correctly from a target density, not just from an approximation; (ii) samples conditional on observing at least one event in an interval; (iii) accomodates user provided random number stream objects; and (iv) is fast. The current version includes one vectorized function for sampling from regular-spaced piecewise constant intensity functions. In future releases we will further optimize execution speed and memory usage.

## 9 Computational details and credits

R 4.3.1 [32] was used for all analyses. Packages **xtable** 1.8.4 [33] and **knitr** 1.45 [34] were used for automatic report generation. Packages **ggplot2** 3.4.4 [35], **ggridges** 0.5.5 [36], and **latex2exp** 0.9.6 [37] were used for plot generation and LaTeXformatting. Packages **nhppp** 0.1.4 [16], **bench** 1.1.3 [38], **rstream** 1.3.7 [39], **otinference** 0.1.0 [40], and **parallel** 4.3.1 were used in the examples and the analyses.

All computations were done on an Apple M1 Max machine with 64 megabytes of random access memory. A preprint of the current paper is in [16]. R itself and all aforementioned packages are available from the Comprehensive R Archive Network (CRAN) at https://CRAN.R-project.org/.

## Supporting information

S1 Appendix Piecewise constant majorizer functions. Algorithm for the automatic generation of piecewise constant majorizer functions.
(PDF)

S2 Appendix Conditional sampling from NHPPPs. Algorithm to sample conditionally on observing at least *m* events in (*a*, *b*].
(PDF)

S1 Code Code to reproduce the exhibits. R code to reproduce the exhibits. Timing results are machine and platform specific.
(TXT)
