# Supplementary material for: The nhppp package for simulating non-homogeneous Poisson point processes in R
Source: PLoS One. 2024 Nov 21;19(11):e0311311. doi: 10.1371/journal.pone.0311311 (PMC11581276; doi:10.1371/journal.pone.0311311)
Supplement: S1 Appendix — Algorithm for the automatic generation of piecewise constant majorizer functions. (PDF) [file pone.0311311.s001.pdf]

S1 Appendix: Piecewise constant majorizer functions

Let $\lambda(t)$ be either a monotonic (and possibly non-continuous) function, or if it is non-monotonic, a K-Lipschitz continuous intensity function, i.e., an intensity function where $|(\lambda(b)-\lambda(a))| \leq K|b-a|$, with K known. Then, Algorithm A finds a piecewise constant majorizing function $\lambda_*(t)$. Starting from a partition of the time interval in time steps (not necessarily equal) it finds an upper bound for λ within each partition.

Algorithm A Pick a majorizing piecewise constant function $\lambda_*(t)$. Partition the interval and find an upper bound for $\lambda(t)$ in each partition.

[h!]

Require:

$\lambda(t)$ is K-Lipschitz in $(a, b]$

Partition interval: $(a, b] = \bigcup_{m=1}^{M}(a_m, b_m]$ ▷ $a = a_1, b_M = b, a_m = b_{m-1}$ $(m > 1)$

$c \leftarrow K$ ▷ Fastest possible slope

if $\lambda(t)$ is monotonic **then** ▷ Then $\sup_{t\in(a_m,b_m]}(\lambda(t)) = \max\big(\lambda(a_m), \lambda(b_m)\big)$

  $c \leftarrow 0$

end if

for $m \in [M]$ **do**:

  $\lambda_m^* \leftarrow \max\big(\lambda(a_m), \lambda(b_m)\big) + c(b_m - a_m)/2$ ▷ Upper bound for $\lambda(t)$ in $(a_m, b_m]$

end for

$\lambda_*(t) \leftarrow \bigcup_{m=1}^{M}\{\big((a_m, b_m], \lambda_m\big)\}$ ▷ Piecewise constant map: $\lambda : (a_m, b_m] \mapsto \lambda_m$

return $\lambda_*(t)$

If $\lambda(t)$ is monotonic, the least upper bound (supremum) is always found at the extremes of the interval and no knowledge of K is required.

The algorithm should be started with a good partitioning of the time interval. In practice, it is generally easy to specify equispaced intervals that are fine enough and impose little computational penalty for the application.

Function `get_step_majorizer()` implements Algorithm A.

```
R> get_step_majorizer(
+    fun = abs, breaks = -5:5, is_monotone = FALSE,
+    K = 1
+  )
```

```
 [1] 5.5 4.5 3.5 2.5 1.5 1.5 2.5 3.5 4.5 5.5
```
