# Supplementary material for: The nhppp package for simulating non-homogeneous Poisson point processes in R
Source: PLoS One. 2024 Nov 21;19(11):e0311311. doi: 10.1371/journal.pone.0311311 (PMC11581276; doi:10.1371/journal.pone.0311311)
Supplement: S2 Appendix — Algorithm to sample conditionally on observing at least m events in (a, b]. (PDF) [file pone.0311311.s002.pdf]

S2 Appendix: Conditional sampling from NHPPPs

Algorithm B is a direct modification of the order statistics algorithm (Algorithm 6 in the main text) to sample conditional on observing m events in $(a, b]$. (The modification is in line 1.)

Algorithm B Modified order statistics algorithm for sampling at least m events from an NHPPP given $\Lambda(t), \Lambda^{-1}(z)$.

[h!]

Require: $\Lambda(t), \Lambda^{-1}(z), t \in (a, b]$ ▷ $\Lambda^{-1}(z)$ possibly numerically
1: $N \leftarrow N \sim \text{TruncatedPoisson}_{N \geq m}\big(\Lambda(b) - \Lambda(a)\big)$ ▷ $(m-1)$-truncated Poisson
2: $t \leftarrow a$
3: $\mathcal{Z} \leftarrow \emptyset$ ▷ $\mathcal{Z}$ is an ordered set
4: **if** N $>$ 0 **then**
5: **for** $i \in [N]$ **do**:
6: $U_i \leftarrow U_i \sim \text{Uniform}(0,\ 1)$ ▷ Generate order statistics
7: $\mathcal{Z} \leftarrow \mathcal{Z} \cup \{\Lambda^{-1}\Big(\Lambda(a) + U_i\big(\Lambda(b) - \Lambda(a)\big)\Big)\}$
8: **end for**
9: $\mathcal{Z} \leftarrow \text{sort}(\mathcal{Z})$
10: **end if**
11: **return** $\mathcal{Z}$ ▷ Up to k earliest points: **return** $\{Z_{(i)} \mid i \leq k\ , Z_{(i)} \in \mathcal{Z}\}$

To sample exactly m points, change line 1 of Algorithm B to

$$N \leftarrow m.$$

To sample up to k earliest points, replace line 11 with in Algorithm B with

$$\textbf{return}\ \{Z_{(i)} \mid i \leq k, Z_{(i)} \in \mathcal{Z}\}.$$
