# Supplementary material for: The nhppp package for simulating non-homogeneous Poisson point processes in R
Source: PLoS One. 2024 Nov 21;19(11):e0311311. doi: 10.1371/journal.pone.0311311 (PMC11581276; doi:10.1371/journal.pone.0311311)
Supplement: Attachment — Submitted filename: Response_to_comments.pdf [file pone.0311311.s004.pdf]

A. Editor comments (EC)

EC.1: *One of the main problems in regression analysis is Multicollinearity; Why not add it to your study? At least add this to future work.*

Reply: This paper is not about regression analysis. Might this comment refer to another paper handled by the editor? If not, please clarify.

EC.2: *Why do you use the "hat" in Equations 1 and 2? The regression model must be without the "hat". Correct it.*

Reply Our Equations 1 and 2 in this paper have no symbols with hats. As above, this paper is not about regression analysis. Please, clarify.

As stated in our letter, it is plausible that we were sent comments for a different paper handled by the editor. If applicable, we are happy to reply to an alternative set of comments by the editor in the next round.

B. Reviewer 1 comments (R1)

R1.1: *The scholarly paper in question present itself as both methodically well-structured and intellectually robust. From the meticulously curated introduction that sets a well-defined premise, down to the scrupulous alignment of its theoretical framework and literature review.*

Reply: Thanks. No reply necessary.

C. Reviewer 2 comments (R2)

R2.1: *I recommend accepting this paper, but after making the following modifications:*

(a) I think that the abstract needs improvement.

(b) I think that some recent papers related to this research should be mentioned.

(c) At the conclusion of this work, the limitations of this research should be mentioned.

Reply: We find it difficult to provide complete replies to Reviewer 2 because the Reviewer's comments are not specific.

(a) It is not clear from the comment what the reviewer finds objectionable about the abstract's text. The abstract is crisp, technically accurate, contextually precise,

and syntactically correct. In addition, it is written in the typical technical style of papers that introduce new software.

Please, provide specific comments for us to address about the abstract.

(b) We introduce a package for the simulation of samples from nonhomogeneous Poisson Point Processes with time varying intensity functions. We are not aware of relevant new research that we have missed. Please provide citations so that we can respond.

Further, this comment is puzzling: We are not introducing new methods. We developed a software and did an extensive numerical and computational-cost[1] study. Given the nature of this paper, what type of relevant research is missing? Is there an `R` package that simulates from NHPPPs over a one-dimensional carrier space that we did not review? We repeated our search but found no new relevant `R` packages.

(c) We introduce a software package in `R`. We have a section on ongoing work and features that we plan to add. What sort of limitations should be added? Is the reviewer complaining about a missing feature? If so, please expand. We will reply whether any absent feature is a design choice, part of future work, or out of scope.

[1] We did the computational cost analysis by recording computation times – not via complexity analysis.
