# Supplementary material for: The nhppp package for simulating non-homogeneous Poisson point processes in R
Source: PLoS One. 2024 Nov 21;19(11):e0311311. doi: 10.1371/journal.pone.0311311 (PMC11581276; doi:10.1371/journal.pone.0311311)
Supplement: Attachment — Submitted filename: response_to_reviewers.pdf [file pone.0311311.s006.pdf]

A. Reviewer 1 comments

R1.1: [No comments]

Reply: [No replies needed]

B. Reviewer 2 comments (R2)

R2.1: *The authors did not make any of the modifications we requested.*

Reply: The comments from Reviewer 2 in the first peer-review round were terse, unclear, and nonspecific. We cannot address comments that we do not understand. We have asked the Reviewer for clarifications to the original comments, or any additional comments, but have received none.

C. Reviewer 3 comments (R3)

R3.1: *The results of this paper are very useful in studies assuming a NHPPP* [sic] *modeling approach with different parametric intensity functions. In many situations, the statistical properties under a classical or a Bayesian approach are verified using simulated data assuming specified parametric intensity functions in NHPPP* [sic]. *This point should be discussed in the introduction section. Although, the importance of the study, the review of the literature on NHPPP* [sic] *should be improved as motivation for the use of the simulation codes (a software package in R) for different structures of NHPPP* [sic] *introduced in the paper.*

In my opinion, the paper could be published after improving the motivation for the need of simulated data assuming a NHPPP [sic] *modeling approach and the inclusion of new references on the use of NHPPP* [sic] *in the introduction section since the use of NHPPP* [sic] *in different applications (environment, epidemiology, economy, engineering) is becoming very popular as observed in the literature. Usually a NHPPP* [sic] *modeling approach is considered assuming different parametric intensity functions, sometimes in presence of one or more change-points, under a Bayesian approach given the difficulties to obtain accurate classical inferences (maximum likelihood estimates and related usual asymptotical results).*

In summary: it is needed to introduce a complete review of the literature on applications of NHPPP [sic] *(new references on the use of NHPPP* [sic] *in different areas) in the introduction section to have better motivation for the study.*

Reply: Following the reviewer's suggestion, we added a paragraph in the introduction to motivate the need for simulating from NHPPPs. It reads (reference numbers are omitted – see text):

"NHPPPs have been used in the simulation analysis of queues in queuing theory and operations research [ref]; hospital operations [ref]; ambulance services [ref]; traffic accidents [ref]; product and network reliability [ref]; and the modeling of cancer [ref], heart disease [ref], and dementia [ref], among other applications [ref]. NHPPPs are used so widely in part because their assumptions are often plausible. For example, when modeling traffic accidents along a road, it may be plausible to assume that individual accidents are independent of each other, but they happen in some locations more often because the probability of an accident depends on local aspects of the road, such as turns, slopes, and propensity for slippery conditions. Similarly, when modeling the impact of screening strategies on colorectal cancer outcomes at the population level, it is probably plausible to assume that, for each person, the emergence of precancerous lesions (adenomas) over a time interval is independent of whether such lesions emerged in other non-overlapping time intervals. In these examples, the intensity of event occurrence over the carrier space (the probability of a traffic accident along a road; and the probability that an adenoma will emerge in a person's colon at different ages) is captured by the NHPPP's intensity function. An NHPPP can model complicated event patterns using intensity functions that vary over the carrier space (e.g., length of road, time)."

D. Reviewer 4 comments (R4)

R4.1: *The proposed manuscript is good and has some merit, but it is not adequate for the* PLOS *journal. The authors should send it to* `R` Journal*, since it shows the performance of a* `R` *package.*

Reply: We have opted to submit to *PLOS ONE* because the manuscript not only introduces the **nhppp** `R` package, but also includes a review of some pertinent theory and a computational accuracy and cost study. This appears concordant with the Journal's scope and aims.

Acceptance and rejection decisions rest with the Editor. As observed by Reviewer 6, *PLOS ONE* has published analogous papers in the past. As of today, the Journal has published at least 86 papers that ostensibly introduce `R` packages.[1]

E. Reviewer 5 comments (R5)

R5.1: *This manuscript presents the* **nhppp** *package for simulating events from one-dimensional non-homogeneous Poisson point processes (NHPPPs) in* `R`*. Related work has already*

[1] Based on the PubMed query `PLOS ONE[so] AND "R package"[ti]` on July 15, 2024.

been published as a preprint. While the manuscript is comprehensive and detailed, it currently resembles a user manual for the toolbox. Before considering acceptance, I recommend that the authors supplement the conclusion with quantitative results, particularly highlighting the advantages of their package over existing toolboxes, such as improvements in efficiency and accuracy, among other metrics.

Reply: Please clarify. Extensive quantitative analyses and comparisons with other `R` packages are already presented in Sections 6 and 7 of the manuscript and in Figures 2 through 7 and Tables 3 through 8. These sections include detailed comparisons with all `R` packages that include functionality to simulate from NHPPPs and reveal that some of the existing packages simulate only approximately.

F. Reviewer 6 comments (R6)

R6.1: *This research is written and formatted in a respectable academic style comparable to studies published in the* `R` Journal. *While I recommend that authors try to publish it in the* `R` Journal, *because it is the best place for this type of research,* PLOS ONE *also accepts these types of studies. Therefore, I recommend accepting publication in* PLOS ONE *after reviewing and ensuring that it conforms to the journal's requirements.*

Reply: Thank you. We decided to submit this paper to *PLOS ONE* based on the Journal's description of its scope. The paper not only introduces a package, but does some review of theory, describes algorithms and intuition for them, includes a numerical accuracy and computational cost study, and does qualitative and quantitative comparisons with other packages. *PLOS ONE* has published papers introducing `R` packages in the past. The Journal has published at least 86 papers that ostensibly introduce `R` packages (PubMed query `PLOS ONE[so] AND "R package"[ti]` on July 15, 2024).
