# Supplementary material for: The nhppp package for simulating non-homogeneous Poisson point processes in R
Source: PLoS One. 2024 Nov 21;19(11):e0311311. doi: 10.1371/journal.pone.0311311 (PMC11581276; doi:10.1371/journal.pone.0311311)
Supplement: Attachment — Submitted filename: Referee report for PLOS ONE-06-2024.pdf [file pone.0311311.s005.pdf]

Referee report for PLOS ONE

Paper: The nhpp package for simulating non-homogeneous Poisson point processes in R.

The results of this paper are very useful in studies assuming a NHPP modeling approach with different parametric intensity functions. In many situations, the statistical properties under a classical or a Bayesian approach are verified using simulated data assuming specified parametric intensity functions in NHPP. This point should be discussed in the introduction section. Although, the importance of the study, the review of the literature on NHPP should be improved as motivation for the use of the simulation codes (a software package in R) for different structures of NHPP introduced in the paper.

In my opinion, the paper could be published after improving the motivation for the need of simulated data assuming a NHPP modeling approach and the inclusion of new references on the use of NHPP in the introduction section since the use of NHPP in different applications (environment, epidemiology, economy, engineering) is becoming very popular as observed in the literature. Usually a NHPP modeling approach is considered assuming different parametric intensity functions, sometimes in presence of one or more change-points, under a Bayesian approach given the difficulties to obtain accurate classical inferences (maximum likelihood estimates and related usual asymptotical results). In summary: it is needed to introduce a complete review of the literature on applications of NHPP (new references on the use of NHPP in different areas) in the introduction section to have better motivation for the study.
